# Utilization of Pharmaceutical Technology Methods for the Development of Innovative Porous Metasilicate Pellets with a Very High Specific Surface Area for Chemical Warfare Agents Detection

**DOI:** 10.3390/pharmaceutics13111860

**Published:** 2021-11-04

**Authors:** Jiří Zeman, Sylvie Pavloková, David Vetchý, Adam Staňo, Zdeněk Moravec, Lukáš Matějovský, Vladimír Pitschmann

**Affiliations:** 1 Department of Pharmaceutical Technology, Faculty of Pharmacy, Masaryk University, Palackého tř. 1946/1, 612 00 Brno, Czech Republic; zemanj@pharm.muni.cz (J.Z.); vetchyd@pharm.muni.cz (D.V.); 507221@mail.muni.cz (A.S.); 2 Department of Chemistry, Faculty of Science, Masaryk University, Kotlářská 2, 611 37 Brno, Czech Republic; hugo@chemi.muni.cz; 3 Faculty of Environmental Technology, University of Chemistry and Technology, Technická 5, 166 28 Prague, Czech Republic; lukas.matejovsky@vscht.cz; 4 Oritest Spol. s r.o., Čerčanská 640/30, 140 00 Prague, Czech Republic; pitschmann@oritest.cz; 5 Faculty of Biomedical Engineering, Czech Technical University in Prague, Sítná 3105, 272 00 Kladno, Czech Republic

**Keywords:** metasilicate, volatile substance, porous pellets, BET method, detection tube, extrusion, spheronization, chemical warfare agent, phosgene

## Abstract

Pharmaceutical technology offers various dosage forms that can be applied interdisciplinary. One of them are spherical pellets which could be utilized as a carrier in emerging second-generation detection tubes. This detection system requires carriers with high specific surface area (SSA), which should allow better adsorption of toxic substances and detection reagents. In this study, a magnesium aluminometasilicate with high SSA was utilized along with various concentrations of volatile substances (menthol, camphor and ammonium bicarbonate) to increase further the carrier SSA after their sublimation. The samples were evaluated in terms of physicochemical parameters, their morphology was assessed by scanning electron microscopy, and the Brunauer–Emmett–Teller (BET) method was utilized to measure SSA. The samples were then impregnated with a detection reagent o-phenylenediamine-pyronine and tested with diphosgene. Only samples prepared using menthol or camphor were found to show red fluorescence under the UV light in addition to the eye-visible red-violet color. This allowed the detection of diphosgene/phosgene at a concentration of only 0.1 mg/m^3^ in the air for samples M20.0 and C20.0 with their SSA higher than 115 m^2^/g, thus exceeding the sensitivity of the first-generation DT-12 detection tube.

## 1. Introduction

Pharmaceutical technology methods are often based on principles transferred from other industries, but they are improved and more accurate because drugs have to meet much stricter quality standards. One of these methods, originally from the food industry, is extrusion/spheronization [1]. This method produces highly spherical particles called pellets with a uniform shape and particle size distribution [2]. Such pellets have suitable properties for drug sorption and subsequent controlled release [3]. This unique ability can also be used in other scientific fields, for example, in analytical chemistry, where detection reagents can be adsorbed on the surface of the pellets and subsequently used for detection purposes [4,5,6]. In practice, in some countries, they are used as carriers in detection tubes, which are part of the equipment of armies and rescue services and are mainly used to detect chemical warfare agents (CWAs), as will be discussed in more detail below.

The development of CWAs began mainly during the First World War (WWI) and they encompass a wide range of chemical weapons that can be divided into 4 generations. Among the 1st generation belong choking agents (e.g., phosgene, diphosgene), blood agents (e.g., hydrogen cyanide) and blister agents (e.g., mustard gas, lewisite), some of which were used during the WWI, while the 2nd, 3rd and 4th generations are formed by nerve agents (e.g., tabun, sarin, soman, VX), binary nerve agents (e.g., GB-2, VX-2) and NOVICHOK (e.g., A-230, A-232 or A-234) [7,8,9,10]. The first nerve agents appeared before the WWII, but fortunately, they were not used during the war. However, it happened later during the Iran–Iraq war in the 1980s [11], then by terrorists in Tokyo Metro 1995 [12], multiple times in Syria between 2013–2018 [13,14], and for assassinations 2017–2020 [15,16,17]. In addition, other CWAs were also used in various conflicts during the 20th century [18]. It is obvious that the CWAs are currently still used mainly by terrorists, assassins or in local and civil wars, despite being banned in 1997 by the Chemical Weapons Convention and their use in armed conflicts is considered a war crime [7,8]. In order to prevent the deaths of the affected and to be able to treat them as quickly as possible, the CWA used must be detected and recognized quickly, sensitively and accurately. For this purpose, a wide range of detectors has been developed [19,20].

One type of such detectors are colorimetric detection tubes (DTs), which are often used in combination with special pumps that allow the use of multiple DTs at once (e.g., CHP-5) [19,21,22,23]. They excel in their simple and relatively cheap design, are easily portable and do not require trained personnel to use, unlike detectors based on, for example, ion mobility spectrometry (IMS), infra-red or Raman spectroscopy, surface acoustic wave (SAW) and other methods [19]. One of the first DTs were developed in 1930s by the German company Dräger, the so-called Dräger-Schröter tubes for the detection of mustard gas. Later, during the WWII, tubes for phosgene, lewisite, cyanogen chloride and hydrogen cyanide appeared in German armaments. This was followed by DTs for nerve agents, which were used along with other types of DTs, for example, in the 1990s in the Gulf War [21]. Nowadays, the DTs continue to be part of the equipment of many armies and rescue services around the world, and their development continues intensively, which is supported by recently published studies on the enzymatic detection of nerve agents [4,5,6,24,25,26]. However, the DTs produced so far were only for a single-use and they utilized older types of detection reagents (e.g., 4-(4-nitrobenzyl)pyridine with N-phenylbenzylamine in the case of phosgene); such DTs can be referred to as first-generation. Nevertheless, the current requirements lead to the need for a new type of DTs that utilize recently developed colorimetric and fluorescent reagents and that would preferably allow continuous or repeated use, so-called second-generation DTs. These new detection reagents have begun to appear in the last decade and often show visible fluorescence. However, they have not yet been tested and optimized for DTs. These reagents include, for example, substances based on rhodamine, pyronine, BODIPY, iminocoumarin and other organic compounds that detect phosgene/diphosgene [27,28,29,30,31]. In order to achieve their highest efficiency, it is also necessary to develop a new type of carrier, ideally in the form of pellets (in the case of DTs), on which the detection reagent is usually impregnated. These carriers must have the required properties, such as high specific surface area (SSA), which enables improved adsorption of substances (especially CWAs and detection reagents) on the carrier surface, spherical shape for optimal colorimetric evaluation, and good mechanical and flow properties that allow the carriers to be easily filled into DTs.

The aim of this study was to prepare innovative carriers in the form of highly porous composite pellets that would have the above-mentioned properties and thus be suitable for interdisciplinary applications, especially for second-generation DTs. To achieve that, a unique substance with a high SSA (300 m^2^/g), a mesoporous, amorphous magnesium aluminometasilicate—Neusilin^®^ US2 was utilized [32,33]. It also has suitable neutral pH, flow enhancing properties and a pure white color, which is suitable for colorimetric detection. As the samples were prepared by extrusion/spheronization as one of the standard methods of pharmaceutical technology, two types of microcrystalline cellulose were also used—Avicel^®^ PH-101 and Avicel^®^ RC-581, which act as a spheronizing agent and filler, but has low SSA of approximately 1.18 m^2^/g compared to Neusilin^®^ US2 [34]. In order to form pores and thus increase the SSA of the prepared samples, volatile substances (VSs) such as menthol (M), camphor (C) or ammonium bicarbonate (AB) were added to the powder mixture in various concentrations, while their particle size was pre-adjusted so as not to exceed 500 µm. Subsequent drying of the samples at 60 °C removed these volatiles, which left pores in the pellet structure, which should result in the increase in the total SSA of the carriers. In addition to mechanical and flow properties, the SSA of the samples was evaluated by the BET method [35] and the particle morphology was analyzed by scanning electron microscopy (SEM). The obtained data were subjected to statistical testing (ANOVA, *t*-test) and multivariate analysis (PCA). In cooperation with Oritest (Prague, Czech Republic), the most promising samples were then newly prepared, evaluated and subsequently impregnated with o-phenylenediamine-pyronine (PY-OPD) as a second-generation detection reagent and tested for the presence of diphosgene/phosgene. This analytical part was described in detail in the recent study [36] and is therefore mentioned only marginally. In addition, such carriers could also be potentially impregnated with any suitable active pharmaceutical ingredient (API) and provide, for example, an immediate release of the API. Such use of porous carriers has been investigated in the study by Cosijns et al. [37], who described the preparation of porous pellets using sodium chloride in combination with Avicel^®^ PH-101, or in the study by Baradari et al. [38], in which porous pellets from calcium phosphate were prepared. In both cases, the porous pellets were subsequently impregnated with ibuprofen (API) and its release from the dosage form was rapid and mainly controlled by diffusion, whereas the SSA of their samples was up to twenty times smaller than the carriers described in this study. The recent patent application (PV 2020–525, Industrial Property Office, Prague, Czech Republic) demonstrates the uniqueness of the newly introduced carriers and the method of their preparation.

## 2. Materials and Methods

### 2.1. Materials

For the preparation of carriers in the form of pellets, menthol racemic, camphor racemic and ammonium bicarbonate (Dr. Kulich Pharma, Hradec Králové, Czech Republic) were used as the pore-forming substances. Neusilin^®^ US2 (magnesium aluminometasilicate, Fuji Chemical Industry, Toyama, Japan) was used as a substance with large SSA and sorption capacity, while Avicel^®^ PH-101 (microcrystalline cellulose, FMC Biopolymer, Wallingstown, Ireland) and Avicel^®^ RC-581 (microcrystalline cellulose and sodium carboxymethylcellulose, FMC Biopolymer, Brussel, Belgium) were used as fillers and spheronization agents. Purified water (Ph. Eur. 10) was used as a wetting agent. All materials were of European Pharmacopoeia grade.

### 2.2. Preparation of Porous Composite Pellets

Table 1 summarizes the composition of 20 types (*n* = 3) of composite pellets with the increasing concentration of VS in the original mixture (sample N0 was used only for comparison in terms of SSA and SEM). Initially, menthol and camphor crystals were each mixed at 17,000 rpm for 30 s using a Microtron MB 800 (Kinematica AG, Malters, Switzerland) to obtain a powder. Menthol, camphor and ammonium bicarbonate powder were subsequently sieved through a 500 µm sieve to ensure a narrower pore size range in the prepared pellets. The powder mixtures according to Table 1 were then homogenized at 1500 rpm for 5 min using a Stephan UMC 5 laboratory mixer (A. Stephan u. Söhne, Hameln, Germany) and later wetted in the same device at a rate of 100 mL/min by purified water at a mass ratio of 1:1.15 (mixture/water). Subsequently, the pellets were prepared using a Pharmex 35T single-screw axial extruder/spheronizer (Wyss and Probst Engineering, Ettlingen, Germany) equipped with a grid with a mesh size of 1.25 mm. The screw speed was set at 60 rpm, the spheronizer speed was 1000 rpm and the spheronization time was 20 min. The prepared samples were dried at 60 °C for 24 h in a Universal Oven UF30 (Memmert, Schwabach, Germany), during which the volatile substances either sublimed (camphor and ammonium bicarbonate) or melted and evaporated (menthol), creating pores in the pellet structure. Using the sieve analysis (according to Ph. Eur. 10), a pellet fraction of 0.80–1.25 mm was obtained and used for further experiments due to its ideal properties for filling into detection tubes [4,24,39].

In cooperation with Oritest, 100 g of each sample (except N0) was impregnated with 50 mL of a 1% solution of PY-OPD (synthesized by the University of Chemistry and Technology, Prague, Czech Republic) in chloroform (Merck, Darmstadt, Germany) and air-dried. The impregnated samples were subsequently filled into glass detection tubes (d = 5 mm) to form a 10 mm long layer and the tubes were hermetically sealed. The samples were then tested with diphosgene, which, unlike phosgene, is in liquid form under normal conditions and can therefore be handled better, while both substances having comparable toxicity. A detailed description of the methodology of impregnation, filling into detection tubes and testing of impregnated samples was described in the related study [36].

### 2.3. Evaluation of Physicochemical Properties

The specific surface area (m^2^/g) of the samples was assessed by nitrogen adsorption/desorption experiments at 77 K on a Quantachrome Autosorb iQ3 porosimeter (Quantachrome Instruments, Boynton Beach, FL, USA). The samples were degassed at 60 °C until the outgassing rate was less than 0.4 Pa/min. The SSA was determined by the multipoint BET method with eleven data points with relative pressures between 0.02 and 0.30.

The hardness (N) of ten randomly selected pellets was evaluated using a C50 Tablet Hardness Tester (Engineering Systems, Nottingham, UK) equipped with a C5 cell and the average with standard deviation was calculated.

The friability (%) of the samples was tested using a TAR10 testing device (Erweka, Langen, Germany). A steel disc vessel was filled with 10 g of the tested pellets and 200 glass spheres (d = 4 mm). The test was performed for 10 min at 20 rpm. The content of the vessel was subsequently sieved and dedusted pellets were weighed. The results were expressed as a percent weight loss of the pellets.

The loss of drying (% w/w) of the samples was measured at 105 °C using a Moisture Analyzer HX204 (Mettler Toledo, Greifensee, Switzerland). The pellets were ground in a mortar with a pestle and approximately 1.0 g was used for each analysis. The results were expressed as a percent weight loss during the drying of the pellets.

The pycnometric density (g/cm^3^) of the pellets (*ρ_p_*) and powder mixtures (*ρ_pm_*) was measured with a helium pycnometer Pycnomatic-ATC (Porotec, Hofheim am Taunus, Germany) according to Ph. Eur. 10. Powder mixtures of the same composition as the pellets were similarly dried at 60 °C for 24 h prior to the evaluation to ensure similar sublimation of VSs.

The interparticular porosity (%) includes open pores of the pellets and unfilled spaces between the pellets. It was calculated for each sample from the values of bulk and pycnometric density of pellets according to Equation (1):

*P_INTER_* = (1 − *ρ*_0_/*ρ_p_*) × 100 (1)

where *P_INTER_* is interparticular porosity (%), *ρ_0_* is bulk density (g/cm^3^) and *ρ_p_* is pycnometric density (g/cm^3^).

The intraparticular porosity (%) includes closed pores inside the pellets and small open pores into which the measuring gas (helium) could not penetrate. It was calculated for each sample from the values of pycnometric density of the pellets and their respective powder mixtures according to Equation (2):

*P_INTRA_* = (1 − *ρ_p_*/*ρ_pm_*) × 100 (2)

where *P_INTRA_* is intraparticular porosity (%), *ρ_p_* is pycnometric density of the pellets (g/cm^3^) and *ρ_pm_* is pycnometric density of the powder mixture with the same composition as the measured pellets (g/cm^3^).

The bulk density (g/cm^3^) includes free air volume between pellets. The volume of 50 g of pellets was measured in a 100 mL graduated cylinder and the bulk density was calculated according to Equation (3):

*ρ*_0_ = *m*/*V*_0_ (3)

where *ρ*_0_ is bulk density (g/cm^3^), *m* is weight (g) and *V*_0_ is volume (cm^3^).

The tapped density (g/cm^3^) reflects a more organized state of particles and therefore, it has higher values than bulk density. The volume of 50 g of pellets was measured in a 100mL graduated cylinder after being tapped 1250 times using an SVM 102 tapping device (Erweka, Langen, Germany) and tapped density was calculated according to Equation (4):

*ρ*_1250_ = *m*/*V*_1250_ (4)

where *ρ*_1250_ is tapped density (g/cm^3^), *m* is weight (g) and *V*_1250_ is volume (cm^3^).

The Hausner ratio describes the flow properties of samples and it is expressed as the ratio between tapped and bulk density according to Equation (5):

*H* = *ρ*_1250_/*ρ*_0_ (5)

where *H* is Hausner ratio, *ρ*_1250_ is tapped density (g/cm^3^) and *ρ*_0_ is bulk density (g/cm^3^).

The sphericity of 200 randomly selected pellets of each composition was evaluated using a stereoscopic microscope NIKON SMZ 1500 (Nikon, Tokyo, Japan) equipped with a 72AUC02 USB camera (The Imaging Source, Bremen, Germany) and NIS-Elements AR 4.0 software (Nikon, Tokyo, Japan).

### 2.4. Scanning Electron Microscopy

All samples were subjected to the examination of morphology by means of SEM. Pellet cross-sections were obtained by capturing a single pellet with tweezers and cutting it with a razor blade. The sample preparation for the microscopic measurement included mounting the specimen on a SEM stub using a conductive carbon double-faced adhesive tape (Agar Scientific, Essex, UK) followed by coating with a 20 nm gold layer using the metal sputtering coating method in the argon atmosphere (Q150R ES Rotary-Pumped Sputter Coater/Carbon Coater, Quorum Technologies, Laughton, UK). The sample images were taken with the utilization of a secondary electron detector (SED) at an accelerating voltage of 3 kV by means of a scanning electron microscope (MIRA3, Tescan Orsay Holding, Brno, Czech Republic).

### 2.5. Data Analysis

In order to draw conclusions on the interrelationships among the pellet/powder characteristics, univariate and multivariate statistical techniques were employed. The effect of the VS type and its concentration on the SSA, hardness, friability, loss of drying, interparticular and intraparticular porosity, bulk and tapped density, Hausner ratio (all mentioned parameters related to pellets) and pycnometric density of pellets and powder mixtures were assessed using the analysis of variance (ANOVA) or *t*-test. Statistical significance of the effects is presented as *p*-values, which are given in the parentheses for each discussed issue throughout the Results and Discussion section (the level of significance was set to the value of α = 0.05). The principal component analysis (PCA) after data standardization was employed to describe the multivariate correlation structure between variables (selected pellet characteristics) and objects (differing in VS type and concentration). In the PCA loadings plot, the lower angle between two variables indicates their positive correlation, while the opposite vectors are negatively correlated. The orthogonality of the two arrows indicates the absence of correlation. The length of the vector expresses the contribution to the sum of explained variability. The positions of the individual samples in the PCA scores plot are associated with the similarity of their properties included in PCA. Samples closely spaced have similar properties, while samples far apart differ from each other. When comparing vector directions in loadings plot and object positions in scores plot, it is possible to deduce the mutual relations between the measured pellet properties and their input characteristics (VS type and concentration). The data analysis was performed by means of software R (version 4.0.1) [40].

## 3. Results and Discussion

In the present study, a new type of carriers in the form of porous composite pellets with a specifically increased surface area was prepared and evaluated using various methods of pharmaceutical technology. To achieve an enhanced SSA of the final product, three different VSs (menthol, camphor and ammonium bicarbonate) with increasing concentration in the powder mixtures were utilized together with a mesoporous magnesium aluminometasilicate (Neusilin^®^ US2) as a substance with high SSA and thus the sorption capacity. During drying of the prepared samples, the VSs sublimed or evaporated from the pellet structure and created pores that increased the SSA. The presence of metasilicate was expected to be essential, which was supposed to be determined by comparing the samples with modified SSA (M7.5–20.0, C7.5–20.0 and AB7.5–20.0) with a blank sample without VSs and Neusilin^®^ US2 (sample N0), and with a second blank sample without VSs but with Neusilin^®^ US2 (sample N). Such pellets have great potential across many scientific fields because, in addition to possible traditional pharmaceutical applications, for example, when the carriers are loaded with a drug (e.g., ibuprofen), they could potentially provide immediate release, as was tested in other studies with different porous pellets [37,38]; they could also be used in analytical detection systems such as detection tubes. In this particular experiment, the modified carriers were intended to be impregnated with a second-generation detection reagent PY-OPD and subsequently used in the glass detection tubes as a means for detecting CWAs, such as phosgene. Although by changing the reagent used, such carriers can also be used to detect other CWAs and toxic substances. Therefore, the main objective of this study was to develop and characterize a new type of carriers for interdisciplinary applications, with their primary use in second-generation DTs, and to determine their most suitable composition with the emphasis on achieving high SSA, while maintaining other suitable physicochemical properties of the samples, such as the appropriate hardness, low friability, high sphericity and good flow properties, as will be discussed in more detail below.

### 3.1. Specific Surface Area and SEM Analysis

**Figure 1:**
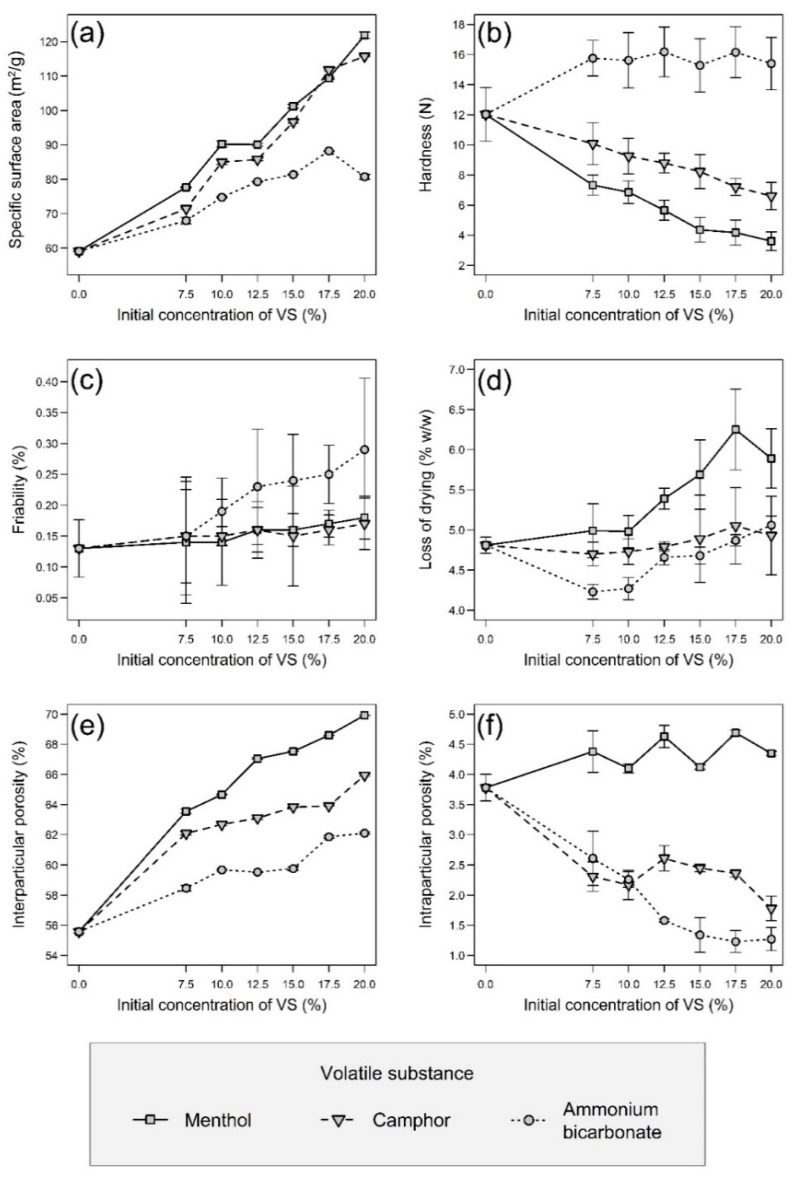
Graphical representation of the results (average values from several measurements) of evaluation of pellets with error bars depending on the volatile substance (VS) used and its concentration in the original powder mixture, where the initial concentration of 0.0% always corresponds to the results of sample N: (**a**) specific surface area, (**b**) hardness, (**c**) friability, (**d**) loss of drying, (**e**) interparticular porosity, (**f**) intraparticular porosity.

**Figure 2:**
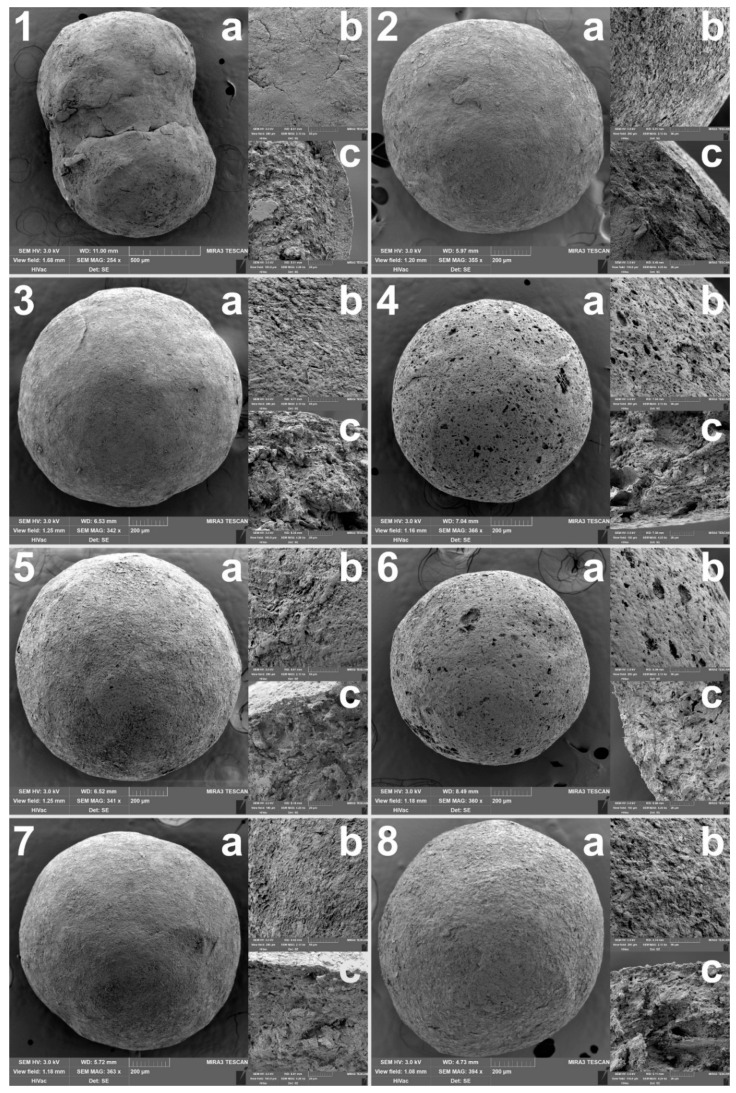
SEM images of selected samples, where **1** is a sample without Neusilin^®^ US2 (N0), **2** is a sample with 25% (w/w) of Neusilin^®^ US2 (N). The remaining samples had a specific surface area modified by the addition of volatile substances, which sublimed during drying: samples **3** (M7.5) and **4** (M20.0) originally contained 7.5% and 20.0% (w/w) menthol, similarly samples **5** (C7.5) and **6** (C20.0) contained camphor and samples **7** (AB7.5) and **8** (AB20.0) contained ammonium bicarbonate. In addition, (**a**–**c**) denote the whole pellet, the surface detail and the cross-section detail, respectively.

The most important evaluated parameter was the specific surface area, as a larger surface would allow better adsorption of both the detection reagent (or possibly API) and CWAs during the detection itself. The SSA was determined by the BET method. The measured values are summarized in Figure 1a, from which it is clear that the utilization of VSs led to a pronounced increase in the SSA, which significantly (*p* < 0.001) increased with their rising concentration in the powder mixtures. Moreover, any type of VS used alone showed a significantly positive effect (*p* < 0.001). When compared to sample N without volatiles (59.12 ± 0.16 m^2^/g), the highest SSA in the case of samples with menthol showed the sample M20.0 (121.87 ± 0.92 m^2^/g), in the case of samples with camphor it was the sample C20.0 (115.74 ± 0.21 m^2^/g), and in the case of samples with ammonium bicarbonate, it was the sample AB17.5 (88.24 ± 0.14 m^2^/g). When converted to the percentage increase in SSA compared to the sample N, it can be observed that the samples M showed 31–106% increase, the samples C 21–96% increase, while the samples AB showed only 15–49% increase. The sample N0 without Neusilin^®^ and VSs was also tested, but even if a sample weight of approximately 2.7 g was used (full vial), the porosimeter was unable to determine its SSA accurately. Thus, it can be stated that the sample N0 was unlike other samples non-porous with a SAA less than 1.0 m^2^/g. Therefore, only by adding Neusilin^®^ US2 to the carrier composition, the SSA of the samples was increased more than 60-fold, which was further increased more than 120-fold than in the case of the sample N0 by adding volatile substances. The presence of pores was confirmed by the SEM analysis, as can be seen in Figure 2. Most pores could be observed in the sample M20.0, which had the highest SSA, whereas the sample N0 with the lowest SSA had a smooth surface with no visible pores. The impregnation of samples with PY-OPD had no negative impact on their SSA and porosity, as was demonstrated in the related study [36].

### 3.2. Hardness and Friability

Other important measured properties of the carriers were hardness and friability, the values of which should be such as to allow further handling of the carriers during their impregnation, filling into detection tubes, storage and transport. The hardness of the pellets has no set limit, but in the case of friability, its values should be less than 1.7% [41]. The results of both measured parameters are summarized in Figure 1b,c. In terms of hardness, both the concentration and the type of VS generally had a significant (*p* < 0.001) effect. When only the concentration was considered, the hardness of samples AB changed insignificantly (*p* = 0.407), while the hardness of samples M and C decreased significantly (*p* < 0.001). The lowest hardness was observed for samples containing menthol, which corresponds to their higher porosity, which made their structure more brittle. In contrast, during the SEM analysis (Figure 2), almost no pores were observed in the samples AB, thus they maintained a similar hardness independent of the ammonium bicarbonate concentration. The friability of all samples was within the given limit, so it can be stated that all samples had suitable mechanical properties. In addition, the VS concentration had an insignificant (*p* > 0.05) effect on friability, while its type had a significant (*p* = 0.001) effect. Samples AB had significantly (*p* = 0.005 and *p* = 0.004) worse friability, probably due to the rougher surface that abraded during the friability test, than samples M and C, which differed insignificantly from each other (*p* = 0.943).

### 3.3. Loss of Drying

A loss of drying generally describes the residual moisture in samples. In the case of the current samples (Figure 1d), it was statistically confirmed that there was a significant effect of the VS type (M > C > AB) and its concentration (both with *p* < 0.001) for samples in general, although the results could be slightly affected by the residual amount of VSs that evaporated during the drying of grounded samples. The loss of drying increased with the rising VS concentration significantly for samples AB and M (both with *p* < 0.001) but insignificantly for samples C (*p* = 0.566). The loss of drying values also corresponded to some extent to the values of hardness and SSA, with a higher loss of drying values being associated with lower hardness and a higher SSA of the samples.

### 3.4. Pycnometric Density

Table 2 shows the pycnometric density values of pellets, and as in the previous cases, its values were generally significantly dependent on the type of substance used (AB > C > M) and its concentration (both *p* < 0.001). The pycnometric density of the dried (at 60 °C for 24 h) powder mixtures of Neusilin^®^ US2 and both types of Avicel^®^ in the same ratios as if the VSs were completely removed from the composition showed in Table 1 increased almost linearly (with R^2^ = 0.9825) from 1.6935 ± 0.0137 g/cm^3^ to 1.8158 ± 0.0086 g/cm^3^ in the direction from 0% to 20% VS concentration. Ideally, the pycnometric density values of the pellets would correspond to that if there were no internal pores or VS residues that tend to decrease the sample density (pure AB has a pycnometric density of 1.5405 ± 0.0058 g/cm^3^, followed by camphor with 1.0048 ± 0.0043 g/cm^3^ and menthol with 0.9639 ± 0.0081 g/cm^3^; on the contrary, Neusilin^®^ US2 has the highest pycnometric density of 2.1779 ± 0.0386 g/cm^3^, followed by Avicel^®^ RC-581 with 1.5647 ± 0.0079 g/cm^3^ and Avicel^®^ PH-101 with 1.5539 ± 0.0079 g/cm^3^). However, from the results of pycnometric density in Table 2 and intraparticular porosity in Figure 1f, it is clear that some VS residues were present in the prepared pellets because their pycnometric density was more lowered and did not increase linearly as in the case of the pure powder mixtures without the VSs. This was most pronounced in samples M, which pycnometric density significantly decreased (*p* < 0.001) with the increasing menthol concentration while having the highest intraparticular porosity of all samples. However, it should be noted that for menthol and camphor, their residual content in the carriers was not detrimental for their possible application mentioned below but instead led to unexpected fluorescence under UV light in the presence of phosgene/diphosgene, which was not observed in the sample N or in samples with ammonium bicarbonate.

### 3.5. Interparticular and Intraparticular Porosity

The pycnometric density values were subsequently used to calculate interparticular and intraparticular porosity which are summarized in Figure 1e,f. Interparticular porosity is more important than intraparticular porosity as it also includes open pores on the surface of the pellets which increase their specific surface area. Interparticular porosity increased significantly with respect to the VS used in the direction AB < C < M and also changed significantly with the increasing concentration of the VS (both *p* < 0.001). Similarly, intraparticular porosity, which encompasses internal closed pores, varied significantly depending on the VS used and its concentration (both *p* < 0.001). Although its values in the case of samples AB, in contrast to the results of interparticular porosity, decreased with the increasing concentration of VS, in the case of samples M and C, there was an inconclusive trend, which is obvious in Figure 1f. In general, samples with a higher proportion of intraparticular pores tend to be more brittle, which was confirmed by the hardness results, where samples with menthol showed the lowest values.

### 3.6. Flow Properties of Carriers

The remaining parameters, such as bulk and tapped density, Hausner ratio and sphericity, describe in particular the flow properties of carriers. Good flow properties are important for further processing of the carriers, such as impregnation (either with detection reagents or API) and their filling into detection tubes or other final packages. The results of all the above-mentioned parameters are summarized in Table 2. In the case of bulk and tapped density and the Hausner ratio calculated on their basis, the values changed significantly in all cases, both in terms of the VS used and its concentration (*p* < 0.001 in each case). For these three parameters, their values decreased in the order AB > C > M, while with the increasing VS concentration, the values decreased in the case of bulk and tapped density in all samples, and in the case of Hausner ratio in samples M and C, whereas samples AB showed an increasing trend. As for the sphericity, it takes values from 0.0 to 1.0, while particles with sphericity higher than 0.8 are considered to be sufficiently spherical (spherical particles generally have better flow properties) [42]. As can be seen in Table 2, this limit was met by all samples, which can therefore be considered sufficiently spherical, as their values are very close to 1.0. Moreover, a statistically insignificant effect of the VS type and its concentration on sphericity was confirmed (both *p* > 0.05). In summary, samples M showed the best flow properties, while samples AB the worst, which even deteriorated with the increasing concentration of ammonium bicarbonate in the original powder mixtures. The worst flow properties of the samples AB confirmed the above-mentioned assumption of their rougher surface.

### 3.7. Principal Component Analysis (PCA)

**Figure 3:**
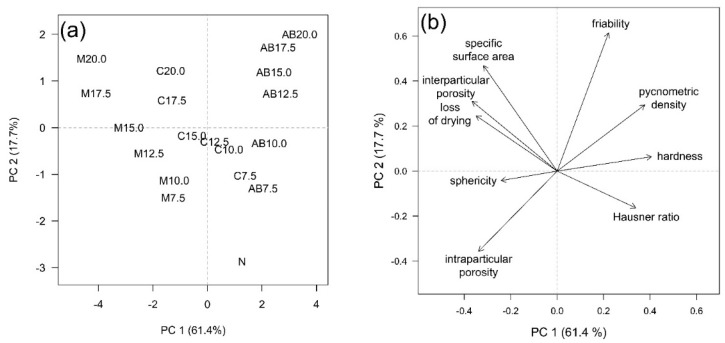
(**a**) PCA scores plot—objects included in the model: pellets differing in type and concentration of the volatile substance. (**b**) PCA loadings plot—variables included in the model: selected pellet characteristics. Bulk and tapped density were excluded from the PCA due to the strong correlation of the respective vectors with the Hausner ratio vector.

In addition, the PCA scores plot (Figure 3a) and loadings plot (Figure 3b) visibly confirm most of the above-mentioned dependencies, while it summarizes the mutual relationships of the VS type used and its concentration with selected carrier properties. The first two principal components together described 79.1% of total variability, which is sufficient [43]. In the scores plot, both dependency trends in data are clearly evident. The distinction between pellets based on the VS type is obvious approximately in the direction of the PC 1 axis, while along the PC 2 axis, the effect of VS presence and concentration is dominant. By comparing the scores plot and loadings plot, it can be deduced that the samples differed depending on the presence and type of the volatile substance (N, M, C, AB) mainly on the basis of parameters such as hardness (AB > C > M), pycnometric density (AB > C > M) and sphericity (statistical testing revealed an inconclusive trend). Depending on the VS concentration used, the samples differed mainly on the basis of the specific surface area and interparticular porosity (both positive correlation with VS concentration). Both mentioned influences then equally (the combination of type of VS and its concentration) played a role in the case of the remaining parameters.

### 3.8. Utilization of Carriers for Phosgene Detection

**Figure 4:**
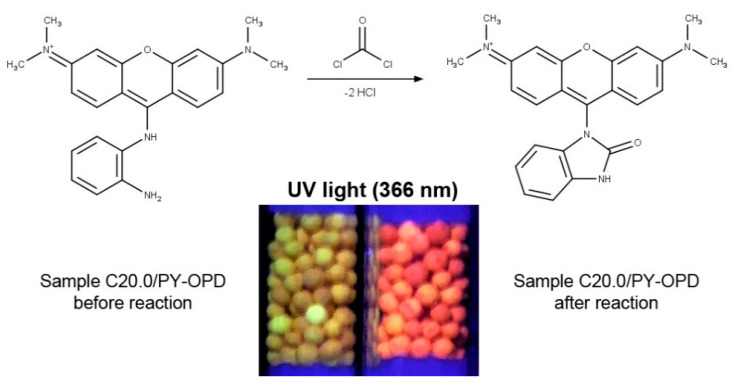
Reaction of o-phenylenediamine-pyronine (PY-OPD) with phosgene and comparison of the impregnated sample C20.0/PY-OPD under UV light at 366 nm before and after the reaction.

**Figure 5:**
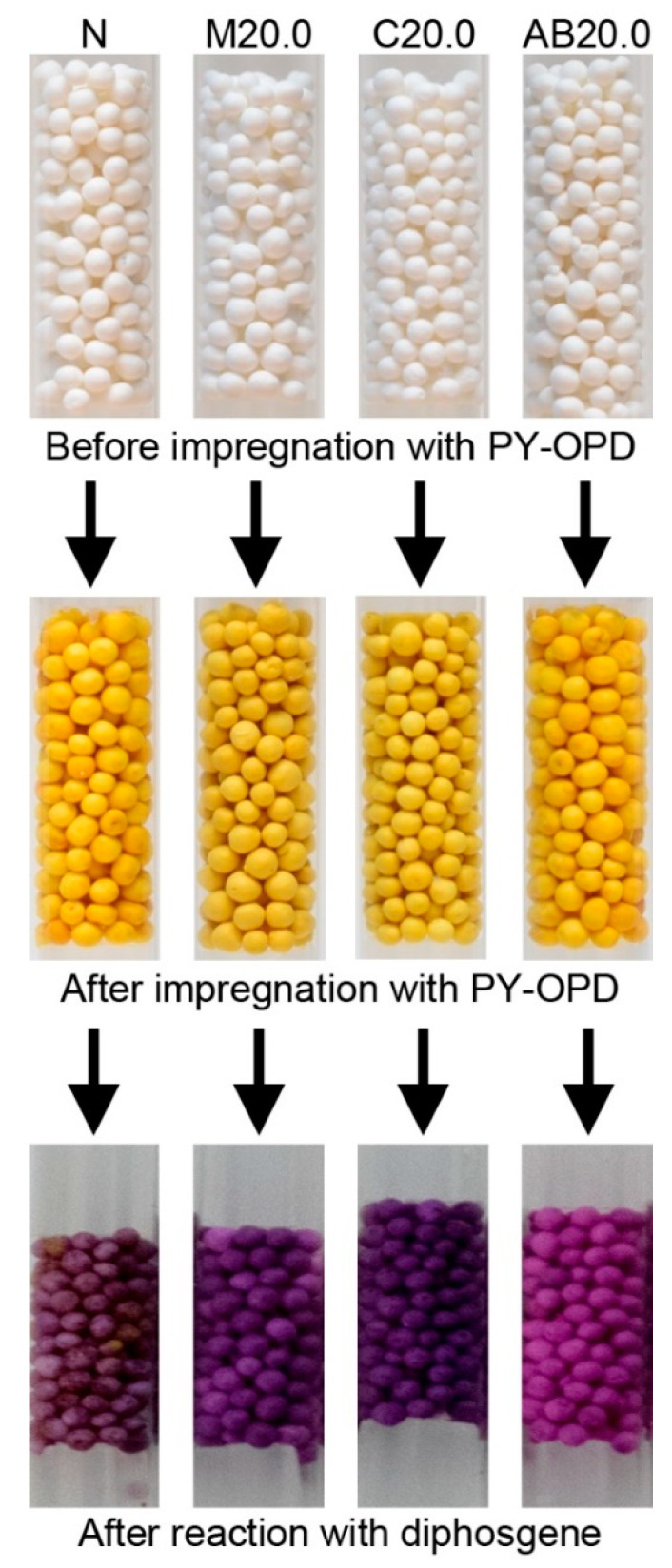
Comparison of the appearance of selected samples before impregnation with the detection reagent PY-OPD, after their impregnation and after their reaction with diphosgene in the visible light spectrum.

Regarding the analytical part, more detailed results of the evaluation of the impregnated pellets, including phosgene/diphosgene sensitivity, selectivity, possible interferences, and sensor stability, were presented in the related, recently published study by Pitschmann et al. [36]. In summary, the mechanism of the reaction of phosgene with PY-OPD is shown in Figure 4, which also depicts the red fluorescence of the sample C20.0/PY-OPD after the reaction with diphosgene/phosgene under UV light at 366 nm compared to the same sample before the reaction. In addition, it was found that although all samples to some extent (sample N and samples AB distinctly less) provided a red-violet color upon the reaction with phosgene/diphosgene, which can be seen in Figure 5, only samples M and C showed fluorescence. Thus, some residual content of menthol and camphor in the carriers seems to be essential. The limit of detection (diphosgene/phosgene) of 0.1 mg/m^3^ was determined for an air sample of 3 dm^3^ using tristimulus colorimetry (UV), which roughly corresponded to less than 2 mg/m^3^ in the case of the naked eye (VIS). For comparison, the currently used detection tubes for phosgene/diphosgene (DT-12, Oritest, Prague, Czech Republic) have a sensitivity of 5 mg/m^3^ [44]. These results represent significant progress in the detection of CWAs using simple, inexpensive, but sensitive and easy-to-operate detection tubes, which was achieved by using the second-generation detection reagent and innovative carriers with a modified, very high specific surface area.

## 4. Conclusions

In this study, a new unique type of porous composite carriers in the form of pellets with a very high specific surface area was developed. This was achieved using the pharmaceutical technology method of extrusion-spheronization. It utilized mesoporous metasilicate Neusilin^®^ US2 as a high specific surface area sorbent, in combination with volatile substances, the sublimation of which at elevated temperatures further increased the SSA of the prepared samples. Comparison of such modified samples with samples without volatile substance and without metasilicate and volatile substance showed up to a 120-fold increase in the SSA of the new carriers while maintaining suitable hardness, low friability, and good flow properties. The use of menthol proved to be the most suitable, followed by camphor. In addition, the samples initially containing these two substances, after impregnation with the second-generation detection agent PY-OPD, showed red fluorescence after the reaction with diphosgene under UV light, which did not occur in the control sample N or samples AB. The determined detection limit then significantly exceeded the limit of the currently used detection tubes. Such carriers can also find application not only in various detection systems, where other CWAs and other toxic substances can be easily detected by changing the detection reagent, but they could also potentially be used as a drug carrier with a wide range of possible applications, especially for immediate release dosage forms, as was discussed in the article.

## Figures and Tables

**Table 1:** Composition of composite pellets (% w/w).

Sample	Menthol	Camphor	Ammonium Bicarbonate	Neusilin^®^ US2	Avicel^®^ PH-101	Avicel^®^ RC-581
N0	0.0	0.0	0.0	0.0	80.0	20.0
N	0.0	0.0	0.0	25.0	60.0	15.0
M7.5	7.5	0.0	0.0	25.0	54.0	13.5
M10.0	10.0	0.0	0.0	25.0	52.0	13.0
M12.5	12.5	0.0	0.0	25.0	50.0	12.5
M15.0	15.0	0.0	0.0	25.0	48.0	12.0
M17.5	17.5	0.0	0.0	25.0	46.0	11.5
M20.0	20.0	0.0	0.0	25.0	44.0	11.0
C7.5	0.0	7.5	0.0	25.0	54.0	13.5
C10.0	0.0	10.0	0.0	25.0	52.0	13.0
C12.5	0.0	12.5	0.0	25.0	50.0	12.5
C15.0	0.0	15.0	0.0	25.0	48.0	12.0
C17.5	0.0	17.5	0.0	25.0	46.0	11.5
C20.0	0.0	20.0	0.0	25.0	44.0	11.0
AB7.5	0.0	0.0	7.5	25.0	54.0	13.5
AB10.0	0.0	0.0	10.0	25.0	52.0	13.0
AB12.5	0.0	0.0	12.5	25.0	50.0	12.5
AB15.0	0.0	0.0	15.0	25.0	48.0	12.0
AB17.5	0.0	0.0	17.5	25.0	46.0	11.5
AB20.0	0.0	0.0	20.0	25.0	44.0	11.0

Note: In the sample designation, N stands for Neusilin, M—menthol, C—camphor, AB—ammonium bicarbonate and 7.5–20.0 is the concentration (% w/w) of the relevant volatile substance in the powder mixture.

**Table 2:** Selected physicochemical properties of pellets.

Sample	Pycnometric Density (g/cm^3^)	Bulk Density (g/cm^3^)	Tapped Density (g/cm^3^)	Hausner Ratio	Sphericity
N	1.6295 ± 0.0037	0.724 ± 0.003	0.775 ± 0.006	1.071 ± 0.005	0.979 ± 0.016
M7.5	1.6349 ± 0.0059	0.596 ± 0.003	0.628 ± 0.003	1.053 ± 0.003	0.983 ± 0.013
M10.0	1.6280 ± 0.0012	0.575 ± 0.001	0.612 ± 0.002	1.063 ± 0.004	0.981 ± 0.014
M12.5	1.6199 ± 0.0031	0.534 ± 0.002	0.562 ± 0.002	1.052 ± 0.004	0.980 ± 0.016
M15.0	1.6211 ± 0.0006	0.526 ± 0.000	0.555 ± 0.002	1.054 ± 0.003	0.984 ± 0.012
M17.5	1.6137 ± 0.0008	0.507 ± 0.001	0.524 ± 0.002	1.035 ± 0.003	0.983 ± 0.014
M20.0	1.6171 ± 0.0006	0.486 ± 0.001	0.496 ± 0.001	1.020 ± 0.000	0.981 ± 0.024
C7.5	1.6655 ± 0.0042	0.632 ± 0.003	0.677 ± 0.003	1.072 ± 0.005	0.977 ± 0.017
C10.0	1.6643 ± 0.0041	0.621 ± 0.002	0.660 ± 0.002	1.063 ± 0.003	0.977 ± 0.017
C12.5	1.6627 ± 0.0035	0.613 ± 0.000	0.647 ± 0.002	1.054 ± 0.004	0.978 ± 0.020
C15.0	1.6554 ± 0.0011	0.599 ± 0.002	0.634 ± 0.001	1.058 ± 0.002	0.983 ± 0.016
C17.5	1.6559 ± 0.0010	0.598 ± 0.002	0.626 ± 0.002	1.048 ± 0.004	0.984 ± 0.015
C20.0	1.6683 ± 0.0035	0.568 ± 0.003	0.594 ± 0.002	1.046 ± 0.007	0.985 ± 0.016
AB7.5	1.6999 ± 0.0078	0.706 ± 0.003	0.746 ± 0.000	1.057 ± 0.004	0.983 ± 0.014
AB10.0	1.7027 ± 0.0023	0.687 ± 0.003	0.728 ± 0.003	1.061 ± 0.004	0.982 ± 0.016
AB12.5	1.7044 ± 0.0004	0.690 ± 0.005	0.735 ± 0.005	1.066 ± 0.008	0.980 ± 0.016
AB15.0	1.7062 ± 0.0050	0.687 ± 0.007	0.723 ± 0.003	1.053 ± 0.011	0.979 ± 0.015
AB17.5	1.7100 ± 0.0031	0.652 ± 0.002	0.696 ± 0.003	1.067 ± 0.008	0.978 ± 0.021
AB20.0	1.7135 ± 0.0033	0.649 ± 0.004	0.706 ± 0.003	1.087 ± 0.008	0.976 ± 0.029

## Data Availability

Data are contained within the article.
